# Extended thymectomy with blood vessel resection and reconstruction improves therapeutic outcome for clinical stage III thymic carcinoma patients: a real-world research

**DOI:** 10.1186/s13019-020-01316-7

**Published:** 2020-09-25

**Authors:** Lei Liu, Jiaqi Zhang, Guige Wang, Chao Guo, Yeye Chen, Cheng Huang, Shanqing Li

**Affiliations:** Department of Thoracic Surgery, Peking Union Medical College Hospital, Chinese Academy of Medical Sciences and Peking Union Medical College, Shuaifuyuan No. 1, Wangfujing Street, Dongcheng district, Beijing, P.R. China

**Keywords:** Thymic carcinoma, Mediastina, Surgery, Vessel reconstruction, Prognosis

## Abstract

**Objectives:**

We examine the therapeutic efficacy of extended thymectomy with blood vessel resection and reconstruction in thymic carcinoma patients with great vessel invasion.

**Methods:**

In total 26 patients diagnosed as clinical stage III thymic carcinoma with severe great vessel invasion were enrolled in this retrospective study. Among these patients, 14 cases received adjuvant chemo- and radiotherapy (non-operation subgroup, NOG), the other 12 patients received extended thymectomy with vessel resection and reconstruction followed by the adjuvant treatment (operation subgroup, OG).

**Results:**

All surgical procedures went smoothly with no perioperative death. R0 resection was obtained in all surgical cases, and we also observed a lymph node metastasis rate of 38.8%. The overall survival (OS) was 34 months for the whole cohort, 48 and 26 months for the OG and NOG respectively (*p* = 0.013). The median disease metastasis free survival (DMFS) was 47 months for the OG and 18 months for the NOG (*p* = 0.019).

**Conclusion:**

Extended thymectomy with vessel resection is feasible for patients with clinical stage III thymic carcinoma. Surgery significantly improves the overall survival and the prognosis of clinical stage III thymic carcinoma.

## Introduction

Thymic tumor is one of the most common neoplasms in anterior superior mediastinum, which accounts for 20% of all mediastinal tumors [1]. In adults, about 50% of anterior mediastinum tumors are thymic tumors. Previous studies have revealed its association with Epstein-Barr virus infections [2, 3]. However, thymic carcinomas are rare and only represent 0.2 to 1.5% of total human malignancies [4]. Pathological subtypes of thymic malignant tumors are generally defined as low- and high-grade. Specifically, low-grade thymic malignancies include squamous cell carcinoma, muco-epidermoid carcinoma, and basaloid carcinoma, whereas lymphoepithelioma-like carcinoma, small cell carcinoma, sarcomatoid tumors, clear cell carcinomas, and undifferentiated tumors belong to the high-grade thymic tumor category [5].

Thymic carcinoma patients rarely exhibit obvious clinical manifestations; therefore, these patients are usually unfavorably identified and diagnosed. Only about one third of thymic malignancy patients develop local symptoms or systemic symptoms [6]. Clinically, the optimal treatment for thymic tumors is surgical operation [7]. However, because of the complexity of anatomical features and vascular distribution in the mediastinum, especially when great vessels are invaded, such surgeries are very challenging to perform, and, in many cases, the tumor is unresectable. All these factors strongly contribute to the poor prognosis for thymic carcinoma. Here, we review thymic carcinoma patients that showed severely invaded great vessels of mediastinum in our center during the past 20 years, and evaluate the efficacy of extended thymectomy with vessel resection and reconstruction for thymic carcinoma patients with great vessel invasions. This study focused on the clinical III stage thymic carcinoma patients with great vessel invasion and analyzed the significance of extended thymectomy with great vessel resection and reconstruction for prognosis.

## Materials and methods

### Patients

We reviewed 292 cases of thymic malignancy diagnosed and treated in our center from 1997 to 2017. Of the above patients,18 were stage I,22 were stage II,65 were stage III and 187 were stage IV. In total, 26 patients diagnosed as clinical stage III thymic malignant tumors by Masaoka stage system with only severe great vessel invasion were selected for this study. 14 of these patients were treated with regular chemo- and radiotherapy and allocated to the non-operation subgroup (NOG); the other 12 patients, who were assigned to the operation subgroup (OG), underwent extended thymectomy with vessel resection and reconstruction as well as routine therapy. The preoperative preparation included enhanced chest CT examination, cardiopulmonary function test and elimination of stage IV thymic carcinoma. We assess resectability by enhanced chest CT, and if the mass was considered to be completely resectable, no biopsy was performed before operation.

### Procedures for extended thymectomy with vessel resection and reconstruction

Following general anesthesia and endotracheal intubation, median sternotomy incision was performed for all the patients in the operation group. In line with preoperative imaging examination, we observed that tumors grew invasively and displayed unclear boundaries with surrounding tissue. Rather than attempting to separate the tumor from the great vessels completely, we decided to perform vascular resection and reconstruction. The operation strategy was to dissociate tumor tissue with invaded vessels preferentially prior to carrying out vessel resection and reconstruction. Tumor and thymic tissues were firstly isolated with the invaded innominate vein or superior vena cava (SVC) followed by blocking the distal end and proximal part of the invaded vessel at the safe edge where the tumor did not invade. When left innominate vein (LIV) was invaded and resected, different approaches were applied for vessel reconstruction, such as LIV- auricula dextra, LIV-atrium dextra and LIV-SVC. If the right innominate vein (RIV) was resected, the major approach for reconstruction was RIV-SVC, except for 1 patient, who underwent RIV- auricula dextra reconstruction. The diameter of the graft ranged in size from 8 to 14 mm, and the choice was decided according to personalized situation during the surgery and 2–4 mL heparin was injected intravenously during bypass grafting in all patients. Polytetrafluoroethylene (PTFE) material was used for vessel reconstruction and autologous pericardium was applied to patch great vessel walls. The great vessels were reconstructed with 4–0 or 5–0 prolene by continuous eversion suture, before which 125 U/mL heparin saline was used to immerse vascular graft prior to vessel reconstruction. If bilateral innominate veins were invaded, LIV was resected and reconstructed preferentially. The SVC blocking time of all the cases were < 40 min, and mostly < 30 min.

### Postoperative treatment

Subcutaneous injection of heparin was administrated to the patients early post-operation according to the severity of chest drainage. For patients with head and neck edema, oral diuretics were given as treatment. In particular, warfarin was given to all patients in the non-operation group as an anticoagulant for 2 months to maintain the international normalized ratio (INR) of prothrombin time of blood coagulationbetween 1.8 to 2.5 and to prevent thrombosis or bleeding complications. The follow-up examination was carried out every 6 months and consisted of CT scans for chest and abdomen to monitor tumor recurrence or metastasis.

For the patients in the OG, postoperative adjuvant chemotherapy and radiotherapy were performed according to their pathological results and intraoperative conditions. The chemotherapy regimen used in this study was mainly TP (Paclitaxel + Cisplatin) or DDP + VP16 (Cisplatin + Etoposide). Total doses of postoperative radiotherapy were 50Gy in 25 fractions by fixed-field intensity-modulated radiation technology.

### Statistics analysis

Overall survival (OS) is defined as the time from the start of treatment to the date of death or to the date of censoring. Distant metastasis-free survival (DMFS) is defined as the beginning of radiotherapy to the detection of distant metastasis or distant metastasis-related death. OS and DMFS were calculated with the Kaplan-Meier method by using SPSS 24.0 statistical software and compared using the log-rank test. Chi-square test was used to detect differences between groups. *P* value < 0.05 was considered statistically significant.

## Results

### General situation of enrolled patients

All patients’ information, including age, sex, clinical symptoms, pathological characters, and operation status are summarized in Table 1. Of the 26 patients enrolled, 19 were male and 7 were female. The mean age of the patients was 52 years (ranging from 20 to 67 years). The diameter range of tumor was between 3.7 to 16.2 cm and the median diameter of tumor was 6 cm. The chi-square test for differences in basic characteristics between the two subgroups is shown in Table 2. Pathological types of thymic carcinoma tumors include squamous cell carcinoma (14/26), atypical carcinoid (5/26), basaloid carcinoma (2/26) and undifferentiated carcinoma (5/26). In the OG, all the 12 pathological reports showed negative margin of tumor. In addition, a total of 6 patients in the OG had lymph node metastasis, with a lymph node metastasis rate of 38.8% (26/67). There was no statistical difference between the two groups in terms of chemotherapy or radiotherapy regimen.

**Table 1:** General situation of included patients

No.	Gender	Age (yrs)	Symptoms	Diameter (cm)	Pathology diagnose	Operation	Status
1	Male	59	Chest pain	5	Basaloid carcinoma	Y	Stable
2	Male	51	Back pain	7.4	Squamous-cell carcinoma	Y	Dead
3	Female	46	Back pain	5.5	Squamous-cell carcinoma	Y	Stable
4	Male	55	Chest pain	3.7	Atypical carcinoid	Y	Stable
5	Male	40	Asymptomatic	7	Atypical carcinoid	Y	Stable
6	Female	47	Myasthenia gravis	7	Squamous-cell carcinoma	Y	Stable
7	Male	34	Chest distress	5.5	Squamous-cell carcinoma	Y	Stable
8	Male	43	Superior vena cava syndrome	5	Atypical carcinoid	Y	Dead
9	Female	52	Myasthenia gravis	6	Squamous-cell carcinoma	Y	Stable
10	Male	23	Superior vena cava syndrome	7.2	Undifferentiated carcinoma	Y	Dead
11	Male	26	Hypokalemia	5	Undifferentiated carcinoma	Y	Dead
12	Female	52	Asymptomatic	5	Squamous-cell carcinoma	Y	Stable
13	Male	53	Chest pain	12.7	Squamous-cell carcinoma	N	Dead
14	Male	65	Myasthenia gravis	5.8	Squamous-cell carcinoma	N	Dead
15	Male	46	Asymptomatic	4.2	Undifferentiated carcinoma	N	Dead
16	Male	53	Shoulder Pain	6.9	Squamous-cell carcinoma	N	Dead
17	Male	58	Asymptomatic	5.8	Atypical carcinoid	N	Dead
18	Female	62	Myasthenia gravis	6	Squamous-cell carcinoma	N	Stable
19	Female	59	Asymptomatic	9.2	Squamous-cell carcinoma	N	Dead
20	Male	67	Chest pain	6	Undifferentiated carcinoma	N	Dead
21	Male	51	Chest distress	16.2	Squamous-cell carcinoma	N	Stable
22	Male	53	Superior vena cava syndrome	5	Undifferentiated carcinoma	N	Lung metastasis
23	Female	39	Asymptomatic	6.6	Basaloid carcinoma	N	Dead
24	Male	53	Chest pain	8.7	Squamous-cell carcinoma	N	Lung metastasis
25	Male	60	Chest pain	9.4	Squamous-cell carcinoma	N	Dead
26	Male	20	Myasthenia gravis	5.5	Atypical carcinoid	N	Stable

**Table 2:** Chi-square test of two groups of basic characteristics

Characteristics	Total	OG	NOG	*p*
Sex				
Male	19	8	11	0.495
Female	7	4	3	
Age				
< 52 years old	12	8	4	0.052
≥ 52 year old	14	4	10	
Tumor diameter				
< 6 cm	12	7	5	0.249
≥ 6 cm	14	5	9	

### Extended thymectomy with vessel resection and reconstruction

**Fig. 1:**
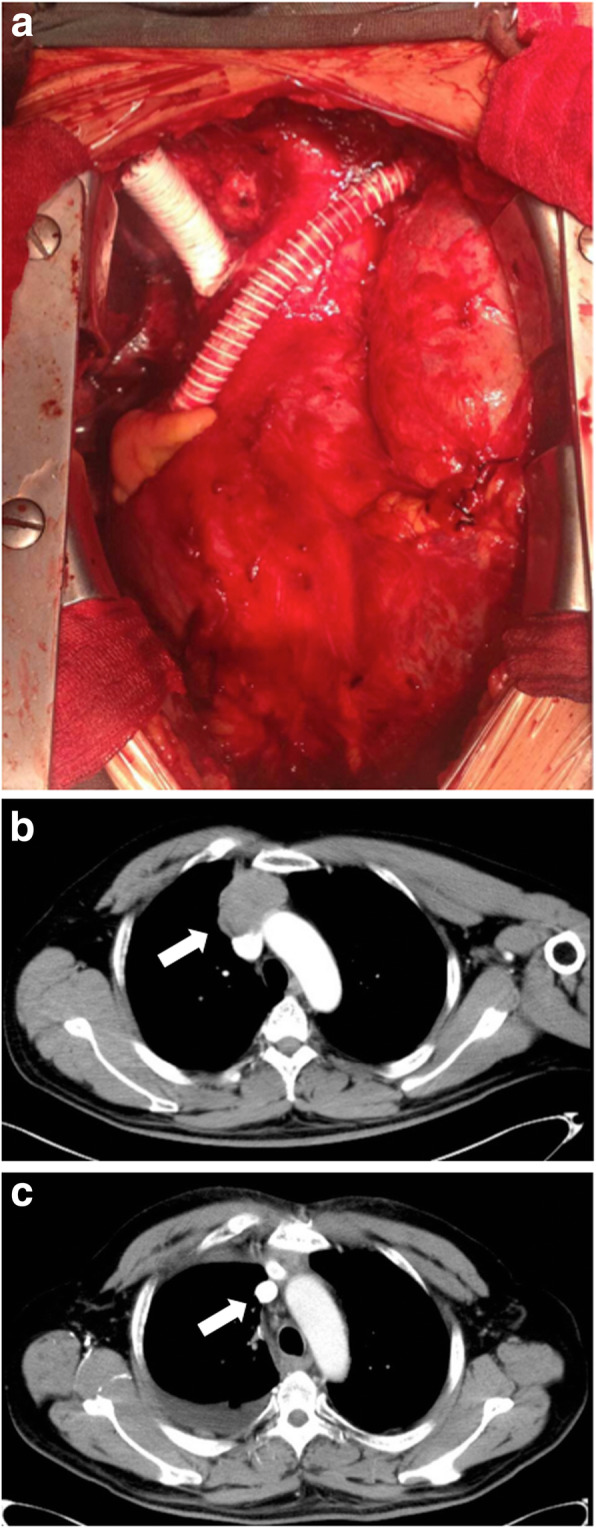
Blood vessel resection and reconstruction. **a** A representative image of the vessel reconstruction after the surgery. **b** CT scan results for a selected patient from the experiment group before the surgery. The arrow indicates the tumor area. **c** CT scan results for a selected patient from the experiment group at the same position after the surgery for six months

Surgical procedures were successful for all patients who received extended thymectomy with great vessel resection and reconstruction, (Table 3). The operation time was between 220 and 405 min, with a mean operation time of 335.83 min. The median postoperative hospital stay was 11.5 days. 7 patients received blood transfusions during the operation, with an average usage of 3 U red blood cells and 266.7 mL plasma, while the mean amount of bleeding was 979.2 mL. 6 cases had multiple vessel reconstructions resulting in a total of 18 vessel replacements. After the surgery, 10 patients received adjuvant therapy, including 6 cases of radiotherapy, 1 case of chemotherapy, and 3 cases of radio-chemotherapy. Of note, there was 1 patient developed a graft thrombus and was re-operated. A representative image of the blood vessel reconstruction is presented (Fig. 1-a), moreover, the typical CT scan results before and after the surgery for 1 selected patient (Fig. 1-b,c) showing not only that the vessel reconstruction was performed satisfactorily but also that no local recurrence is visible.

**Table 3:** Operation Status of OG

Variable	Numerical value
Time of operation, min (range)	335.83 (220–405)
Postoperative hospital stay, day (range)	11.5 (4–46)
Diameter of vessel replaced	
8 mm	13 (72.22%)
12 mm	4 (22.22%)
14 mm	1 (5.56%)
Amount of bleeding, ml (range)	979.17 (200–2500)
Method of vessel reconstruction	
LIV- auricula dextra	6 (33.34%)
LIV- atrium dextra	2 (11.11%)
LIV-SVC	2 (11.11%)
RIV-SVC	7 (38.88%)
RIV- auricula dextra	1 (5.56%)
Resected Vessels	
LIV	4 (33.33%)
RIV	2 (16.67%)
LIV + RIV + SVC	6 (50.00%)
Blood transfusion	
RBC, U (range)	3 (0–9)
Plasma, ml (range)	266.67 (0–1000)

*LIV* left innominate vein, *SVC* superior vena cava, *RIV* right innominate vein

### Prognosis for clinical stage III thymic malignancy patients with great vessel invasion

**Fig. 2:**
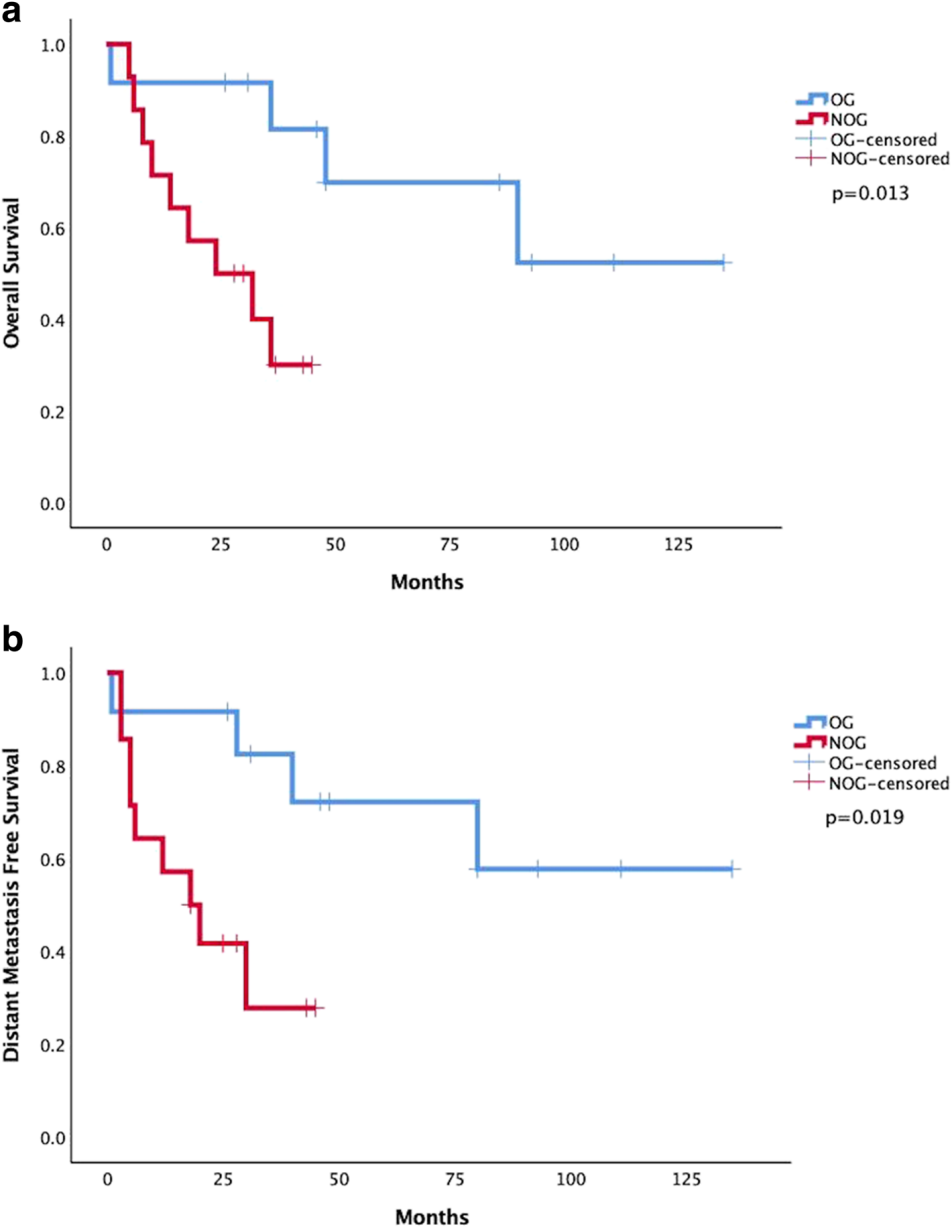
The Comparison of prognosis between control and experiment group. **a** Overall survival (OS) in the control group and experiment group, *P* = 0.013. **b** Distant metastasis free survival (DMFS) in the control group and experiment group, *P* = 0.019

The median follow-up time of patients was 44 months (ranging from 21 to 143 months). For the 14 patients in the NOG, we observed 9 deaths with distant metastasis of different sites was observed before death by the time of the last follow up and 2 patients exhibited lung metastasis. In contrast, among the 12 patients from OG, except for one patient who died of acute respiratory failure 1 month after surgery, 3 patients had died at the time of the last follow up with thoracic cavity, liver or bone metastasis. The other 8 patients from the OG showed no metastasis or local recurrence of the disease. To the date of the last follow up, the survival rate was 66.67% for the OG and 35.71% for the NOG. The median OS of the 26 patients is 34 months (range 1 to 135 months), it is 48 months for the OG and 26 months for the NOG. The overall median DMFS is 28 months while it is 47 months for the OG and 18 months for the NOG. The differences in OS and DFMS between the two subgroups are significant (*p* = 0.013 and *p* = 0.019 respectively) (Fig. 2-a,b).

## Discussion

Although the occurrence is low, thymic tumors are one of the most frequent mediastinal neoplasms, and account for 5% of all thymic malignancies [8]. Venuta et al. have reported that approximately 30% of patients with thymic malignancies are asymptomatic, 40% of them show local symptoms, and only 30% exhibit systemic symptoms [9]. Thus, except for those displaying systemic symptoms such as myasthenia or pure red cell aplasia, many thymic malignancy patients lack clinical manifestations. Therefore, a large number of thymic carcinoma is of relatively late stage at the time of diagnosis. In this study, 17 patients were asymptomatic or had mild clinical symptoms such as chest-back pain and chest tightness, accounting for 65.39% of all patients. Besides, for adhesion to great vessels like SVC and characteristics of aggressive growth of malignant tumors, thymic carcinoma are very difficult to completely remove which leads to a poor prognosis. We retrospectively analysis 26 patients with thymic carcinoma diagnosed as clinical stage III due to great vessel invasion to study the effects of different treatments on prognosis in real-world.

Extended thymectomy was first proposed by Masaoka in 1981, he defined it as the removal of the extra-capsular fat with the intra-capsular thymus gland, for eliminating as much thymic tissue as possible [10]. In 1996, Masaoka consolidated the concept as resection of the anterior mediastinal fat tissue, which includes the thymus; the operation was performed bluntly from pericardium and pleura. In the procedure of external thymectomy he described, adipose tissues around the upper poles of the thymus, around both brachiocephalic veins, and on the pericardium were resected meticulously. If necessary, the pleural cavity was entered. The borders of resection were the diaphragm caudally, the thyroid gland orally, and the phrenic nerves laterally [11]. Thymectomy in now routinely used for the resection of mediastinal tumors [12, 13]. Additionally, extended thymectomy is the most acceptable and efficient therapy for thymic carcinoma [14].

Involvement of the great vessels has been found to be an independent negative prognostic factor [15] and favors the onset of recurrence [16] in thymic carcinoma. Great vessel invasion do not only increases the difficulty and risk of the operation, but can even sometimes completely preclude it. Previous studies have reported on great vessel resection and reconstruction but most of them were case reports [17, 18]. Due to the unsatisfactory effect of radiotherapy and chemotherapy, we did our best to perform surgical treatment for the 12 patients in the study. According to our observations, the most commonly invaded vessel is the LIV due to the proximal anatomical feature. Although the operation went fine, it remains a difficult procedure, as can be appreciated from the amount of bleeding and blood transfused during the operation. After reviewing the procedure, we could conclude that the extended thymectomy with blood vessel resection and reconstruction can be completed following a defined procedure; we therefore propose that the operation could be normalized and popularized.

Previous research showed that a tumor diameter larger than 8 cm is a risk factor for recurrence [19, 20], while complete resection of a tumor is extremely important for improving prognosis [21–23]. The study of Bacha et al. retrospectively analyzed 89 patients who underwent total or subtotal resection of a primary mediastinal tumor and suggested median sternotomy as an excellent surgical approach [24]. To achieve such a goal and assure safety during the surgery, we adopted median sternotomy for all the patients, which allowed vessel resection and reconstruction and ensured the range of extended thymectomy. At present, there is no clear definition of surgical indications in the latest guidelines, whether thymic carcinoma can be operated depends to a large extent on the surgeon’s appreciation. However, in our retrospective study, there was no significant difference in tumor diameter between the two groups (*p* = 0.249). In real clinical practice, tumor diameter will become one of the factors that influence the surgeon decisions, especially on the issue of resectability.

Rajan et al. indicated that resection of the entire tumor by complete thymectomy and removal of all surrounding mediastinal fat as well as surrounding pleura increased the chances of achieving negative surgical margins [1]. Among the 12 thymic carcinoma patients who underwent operation, all of them had a complete tumor resection with grossly and pathologically confirmed negative resection margins. However, due to the large volume of the tumor, we still can’t certainly exclude the possibility of R1 resection. Some researchers have demonstrated that postoperative radiotherapy reduced thymic malignancy recurrence [25]. In order to reduce postoperative relapse and metastasis, 10 patients received adjuvant therapy, including 6 cases of radiotherapy, 1 case of chemotherapy and 3 cases of radio-chemotherapy. A multicenter study based on a Chinese population found that the lymph node metastasis rate of thymic carcinoma could be as high as 25%, and after intentional lymph node dissection or sampling, the lymph node metastasis rate increased significantly [26]. In this study, the rate of lymph node metastasis was higher (38.8%). We believe that this is associated with the higher overall stage of our cohort, but the above data all suggest the importance of lymph node dissection in thymic carcinoma surgery.

Most patients of the OG had a graft diameter of 8 mm, compared to 12 mm or 10 mm vascular prosthesis used in the studies carried out by Bacha et al. [24]*.* and Shintani et al. [27]. There is no standard procedure to determine the diameter of artificial vessels. We decided on the diameter for vascular replacement based on the intraoperative evaluation of the invaded vascular. The diameter of artificial blood vessels in the OG was relatively fine due to the Asians figure. However, we experienced 1 patient who had acute thrombosis after surgery and was re-operated with a 12 mm graft replacement, as the reconstructed vessel might have been too small for the patient. Previous studies suggested systematic administration of heparin before vessel replacement [17, 18, 24], we also gave 2–4 ml heparin during the operation and subcutaneous heparin after the operation to prevent embolism. In addition, oral warfarin was given for 2 months for anticoagulation to maintain the INR between 1.8–2.5. To prevent thrombosis, all grafts were immersed in 125 U/ml heparin saline before vessel reconstruction. To date, with the long-term follow-ups, no severe vascular stenosis in the reconstructed vessels was observed.

Chung et al. reported that the median survival for patients with stage III thymic malignancy incompletely or not resected and invading neighboring organs was 26.1 months [28]. Eng et al. concluded that five-year survival rates for thymic carcinoma were approximately 30 to 50% [29]. Similarly, previous literature reported that the overall 5-year survival rate was 42% for thymic carcinoma patients [24]. In our study, we compared the prognosis between the two subgroups: in OG, 4 patients died and suffered thoracic cavity, liver or bone metastasis by the time of the last follow-up; in contrast, 9 patients died, and 2 patients showed metastasis in the NOG. To the date of the last follow up, the survival rate is 66.67 and 35.71% for the OG and NOG respectively. The median OS of all patients included is 34 months; whereas OS of OG is significantly longer than NOG (48 vs. 26 months, *p* = 0.013). DMFS of OG is also significantly longer than NOG (47 vs. 18 months, *p* = 0.019). The main recurrence pattern of thoracic adenocarcinoma is in situ relapse. Results above suggest that extended thymectomy with vessel resection and reconstruction can significantly improve the prognosis of clinical stage III thymic malignancy with great vessel invasion, and reduce the probability of metastasis.

This study has several limitations. First, due to low incidence of thymic carcinoma, and the study inclusion criteria that were limited to patients with clinical stage III thymic carcinoma, the patient cohort is small. Second, since this is a retrospective analysis, there will be unavoidable selection bias. We had limited the criteria for inclusion to clinical III thymic carcinoma with only vascular invasion to minimize selection bias. Last but not least, this study failed to continue to explore the impact of clinicopathological features, such as tumor diameter, lymph node metastasis, and pathological type, on the prognosis of patients in the OG.

## Conclusion

Thymic carcinoma patients often lack typical clinical manifestations and are therefore in a relatively advanced stage at the time of initial diagnosis. We conducted a real-world research reviewed the data of clinical stage III thymic carcinoma patients who underwent extended thymectomy with blood vessel resection and reconstruction. We consider that it is feasible to carry this operation for patients at this stage, and it can be popularized as a routine operation manner. We found that surgery can not only improve the prognosis of patients at this stage, but also reduce the probability of distant metastasis. Our data also confirmed the high rate of lymph node metastasis in thymic carcinoma patients and propose intentional lymph node dissection or sampling during thymic carcinoma surgery.

## Data Availability

Not applicable.

## Abbreviations

NOG: Non-operation subgroup
OG: Operation subgroup
OS: Overall survival
DMFS: Disease metastasis free survival
SVC: Superior vena cava
LIV: Left innominate vein
RIV: Right innominate vein
PTFE: Polytetrafluoroethylene
INR: International normalized ratio
